# Supplementary material for: In Situ Proteolysis Condition-Induced Crystallization of the XcpVWX Complex in Different Lattices
Source: Int J Mol Sci. 2020 Jan 2;21(1):308. doi: 10.3390/ijms21010308 (PMC6981927; doi:10.3390/ijms21010308)
Supplement: Supplementary file 1 [file ijms-21-00308-s001.pdf]

Supplementary Materials

Table S1. Twin law of the diffraction data of the P3 hexagonal lattice

Operator	Type	Axis	R obs.	Britton Alpha	H Alpha	ML Alpha
∣ -h, -k, l ∣	Merohedral	2-fold	0.425	0.077	0.068	0.022
∣ h, -h-k, -l ∣	Merohedral	2-fold	0.296	0.191	0.198	0.133
∣ -k, -h, -l ∣	Merohedral	2-fold	0.420	0.078	0.070	0.022

Table S2. Contact formation among symmetry molecules in the two lattices

Lattice	Molecule	No. of Contact-forming Residues	Symmetry Operation	Symmetry Molecule	No. of Contact-forming Residues	Interface Area (Å)
Orthorhombic	X	26	x-1/2,-y+1/2,-z	W	18	784.1
	W	17	-x-1,y-1/2,-z-1/2	X	15	475.6
	X	13	x-1/2,-y+1/2,-z	X	13	383.4
	X	11	x-1/2,-y-1/2,-z	V	6	246.4
	X	7	x-1,y,z	W	8	179.2
	V	3	-x-2,y-1/2,-z-1/2	X	7	137.8
	V	5	x-1/2,-y-1/2,-z	W	4	131.3
	X	4	-x-2,y-1/2,-z-1/2	X	6	109
	V	7	x-1/2,-y-1/2,-z	V	4	103.7
	W	4	-x-2,y-1/2,-z-1/2	X	3	103.5
	W	2	-x-1,y-1/2,-z-1/2	W	4	38
	V	2	x,y-1,z	X	1	26.8
	**Total**	**101**			**89**	**2718.8**
Hexagonal	X1	23	-y,x-y-1,z	W1	26	753.7
	W2	19	-y-1,x-y-1,z	X2	24	590.3
	W3	20	-y,x-y,z	X3	21	526

X2	4	x,y,z-1	V2	10	214.8
X3	4	x,y,z-1	V3	7	165.8
V1	4	x,y,z-1	X1	3	81.5
W2	10	x,y,z-1	W1	6	205.3
W3	2	x,y,z-1	V3	4	78.2
W2	2	x,y,z-1	V2	2	12.5
W3	2	-y-1,x-y-1,z+1	X2	3	51.2
X1	2	-y,x-y-1,z	X1	3	28.6
W2	1	x,y,z-1	X1	3	27.4
X2	1	x,y,z-1	W1	1	23.8
**Total**	**94**			**87**	**2005**

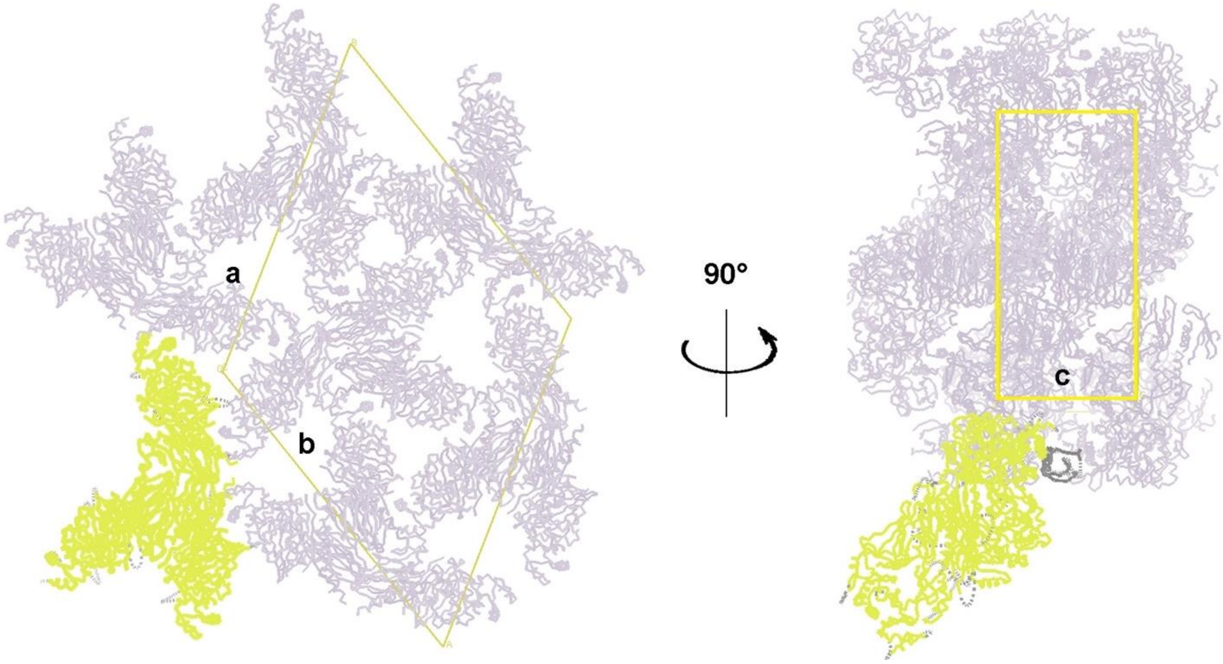

Figure S1. Packing of the XcpVWX complex molecules in the P3 hexagonal lattice in different views. In the dimension of **a** and **b**, molecules form triangular cavities. The side view of the lattice indicates that the packing in this dimension is very tight due to length of **c**.

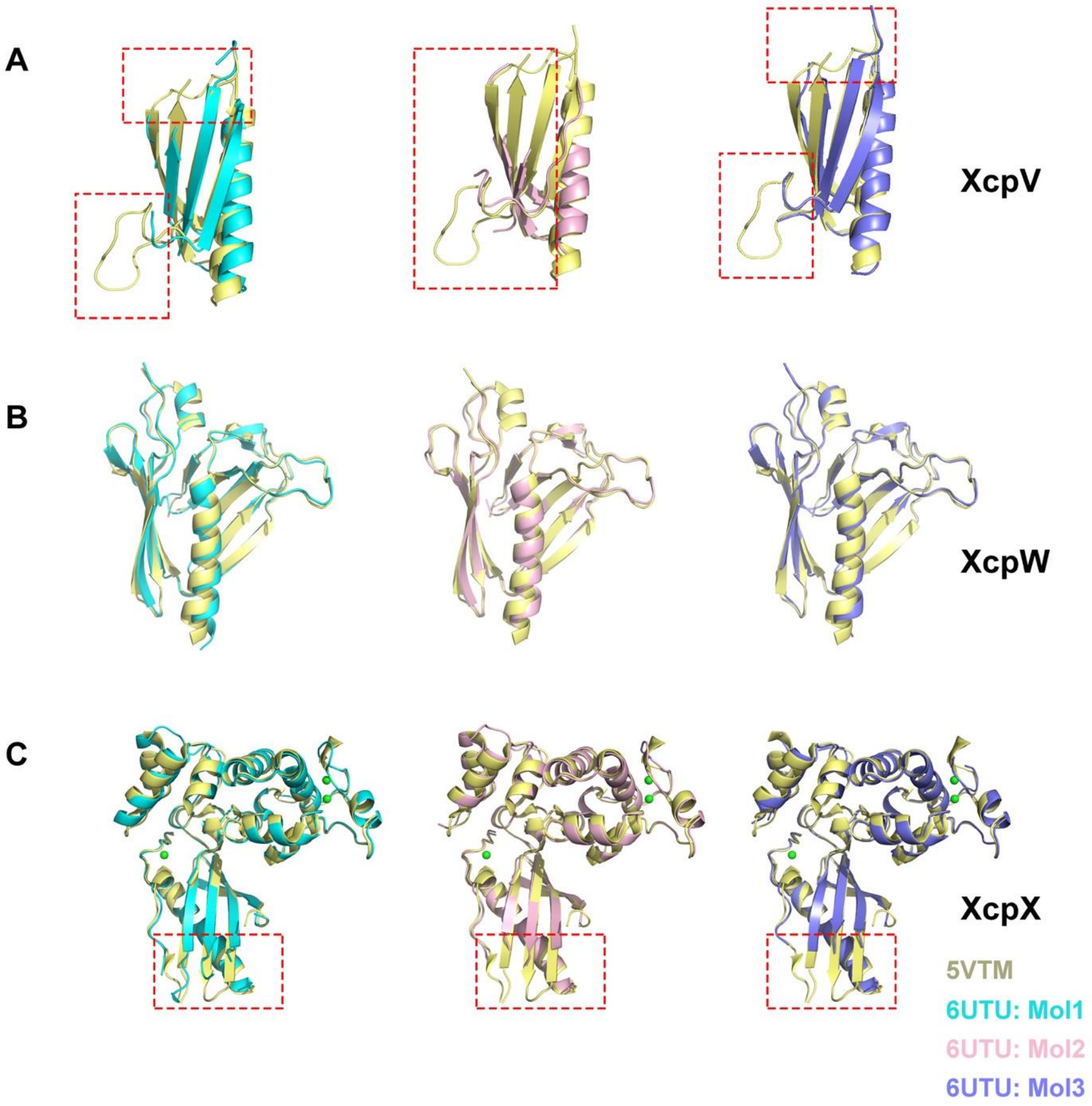

Figure S2. Structure comparison between the complex molecules in the orthorhombic and the hexagonal lattices. Alignment of the structures of XcpV(A), XcpW(B), and XcpX (C) demonstrates the detailed structural differences.

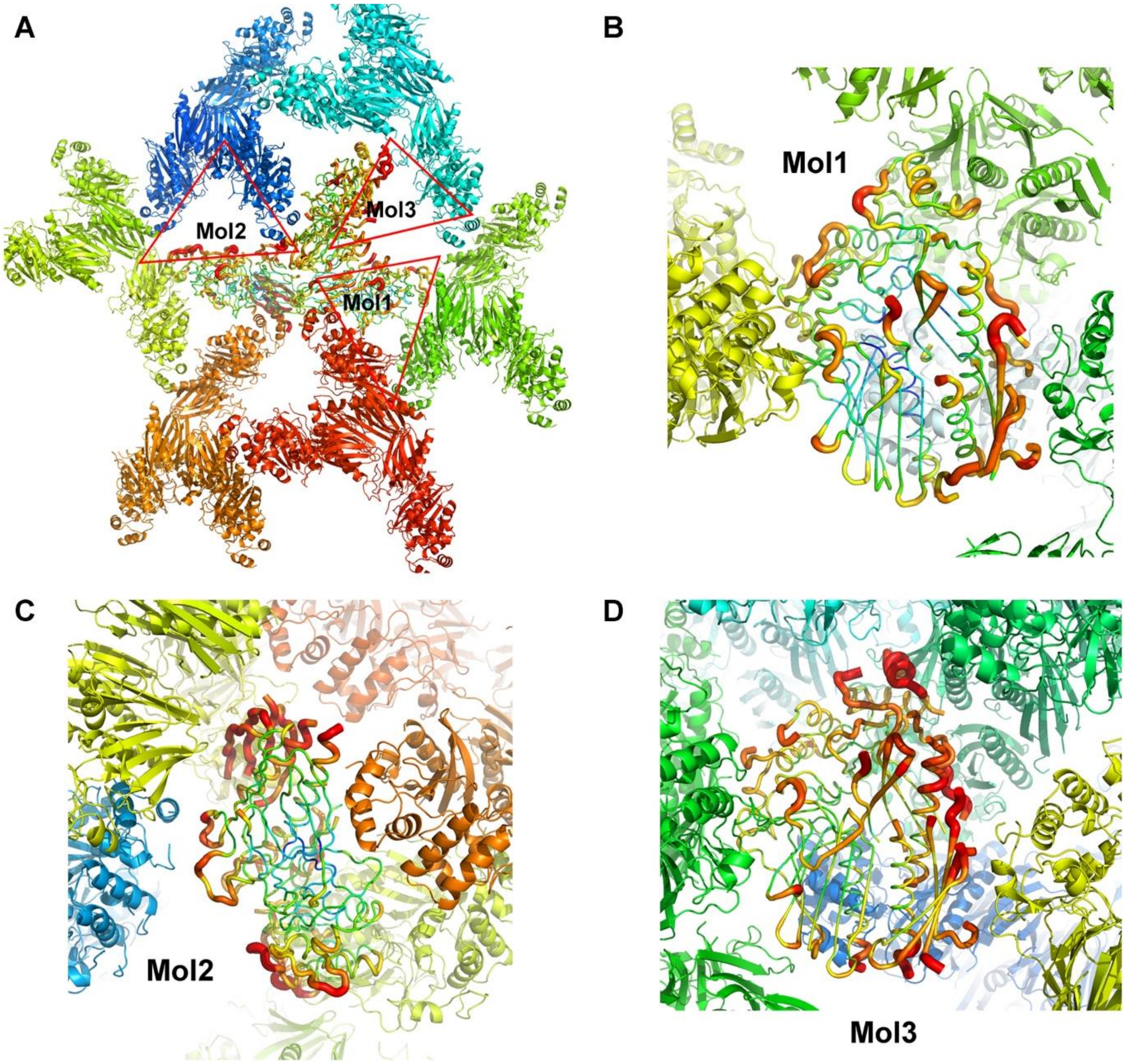

Figure S3. Relationship between ADP and contact formation in the complex molecules in the P3 hexagonal lattice. (A) General view of the ADP distribution of the complex in the lattice**.** Regions with high ADP values are on the sides of the triangles. (B), (C) and (D) Average ADP of individual complex molecule when associating with other adjacent symmetry molecules. It is shown that the space around high-ADP regions are large to accommodate high flexibility and atomic motions. The contacts between molecules formed in these regions are relatively loose and limited.

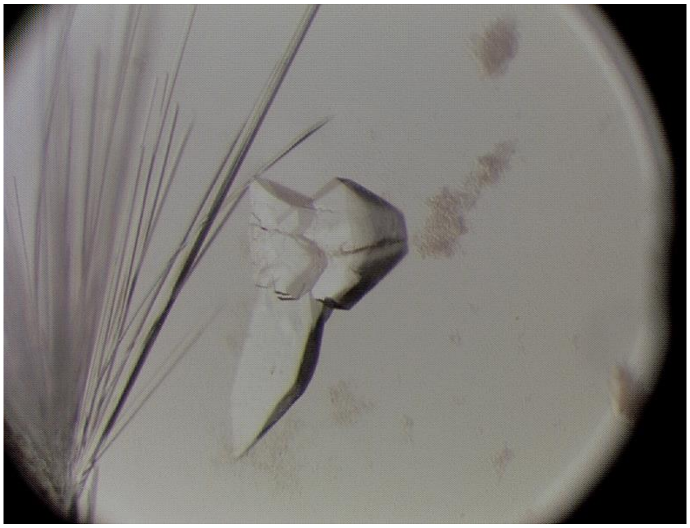

Figure S4. Two forms of the XcpVWX ternary complex crystals are observed in the same crystallization drop. The hexagonal-lattice crystals are in a chunk-like crystal cluster. The orthorhombic-lattice crystals display a form of a rod-like crystal cluster.
